# Psychological outcomes in paediatric major trauma patients who require invasive management: a systematic review

**DOI:** 10.1136/bmjpo-2025-004450

**Published:** 2026-03-19

**Authors:** Owen Hibberd, Caroline Thomas, Sarah Gentle, Sandi Angus, Spyridon Karageorgos, Stephen H Thomas

**Affiliations:** 1 Emergency and Urgent Care Research in Cambridge (EURECA), PACE Section, Department of Medicine, University of Cambridge, Cambridge, UK; 2 Blizard Institute, Queen Mary University of London, London, UK; 3 Georgetown University, Department of Biochemistry, N.W, Washington, DC, USA; 4 School of Medicine, University of Crete, Heraklion, Greece; 5 Pediatric Emergency Department, Aghia Sophia Children's Hospital, Athens, Greece; 6 School of Medicine, European University Cyprus, Nicosia, Cyprus; 7 Department of Emergency Medicine, Beth Israel Deaconess Medical Center, Harvard Medical School, Boston, Massachusetts, USA

**Keywords:** Adolescent Health, Stress Disorders, Traumatic, Acute, Child Psychiatry

## Introduction

 Paediatric major trauma is a leading cause of morbidity and mortality in children, often necessitating invasive management and intensive care.^1 2^ In addition to physical injuries requiring intervention, holistic care for those who have sustained a serious injury should also consider their mental health needs.^1 2^

This study aims to explore whether paediatric major trauma patients needing surgery or interventional radiology have higher odds of adverse psychological outcomes than those managed conservatively (ie, without procedures).

## Methods

This systematic review included paediatric major trauma patients (<16 years) who had invasive management (operation or interventional radiology) compared with conservative management, with data available on the incidence of subsequent psychological disorders. Medline (via Ovid), Embase (via Ovid), PsycInfo (via Ebscohost) and CINAHL (via Ebscohost) were searched from inception to August 2025. Two reviewers assessed each article at each stage of screening, data extraction and risk-of-bias assessment. The full protocol and peer-reviewed search strategy for the study are published online.^3^

There was no specific patient and public involvement in this study. However, an established James Lind Alliance priority-setting partnership, which involved patient co-design, emphasises the psychological aspects of major trauma care for patients and their families.^4^

## Results

**Figure 1:**
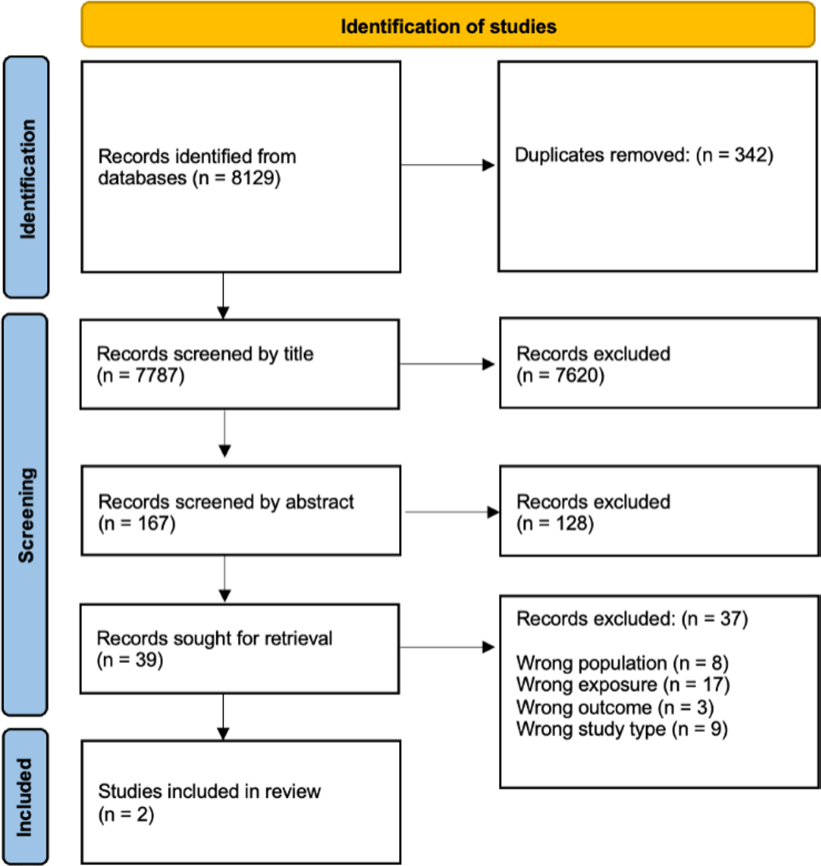
Preferred reporting items for systematic reviews and meta-analyses flow diagram.

The search strategy identified 8129 studies; following screening, 39 full texts were reviewed, and two studies met the inclusion criteria (figure 1).

The characteristics of the two studies identified are shown in table 1.

**Table 1:** Study and patient characteristics

Title	Child anxiety, depression and post-traumatic stress disorder following orthopedic trauma	Acute stress disorder in the paediatric surgical children and adolescents injured during the Wenchuan earthquake in China
Author	Al Zomia *et al*	Liu *et al*
Hospital setting	Yes	Yes
Study type	Retrospective cross-sectional	Survey
Country of treatment	Saudi Arabia	China
Cohort size	100 children	105 children
Gender	67/100 (67%) male	50/105 (47.6%) male
Mean age (±SD)	7.3±3.4 years	11.8±3.8 years
Type of injury	Motor vehicle collision 10/100 (10%) Fall from height 37/100 (37%) During play 44/100 (44%) Other 9/100 (9%)	Buried during earthquake=48/105 (45.7%) Not buried during earthquake=57/105 (54.3%)
Injury severity score	Not reported	Not reported
Prior mental health diagnosis	Family history of psychiatric disease reported in 5/100 (5%) of children. Past medical history not reported.	Excluded prior psychotic illness
Head injury	12/100 (12%)	Not reported
Fatality at the scene	Not reported	18/105 (17.1%) dead or missing relatives
Family member also injured	Not reported	5/105 (4.8%) seriously injured relatives
Death of a family member	Not reported	18/105 (17.1%) dead or missing relatives
Screening tools used for adverse psychological outcomes	Revised Children’s Anxiety and Depression Scale-25 and Combined Child Trauma Screen for PTSD	Acute Stress Disorder scale
Incidence of adverse psychological outcomes	5/100 (5%) clinically significant PTSD	57/105 (54.3%) ASD
Operation	70/100 (70%) had an operation. p=0.049 for clinically significant PTSD	83/105 (79%) had an operation p<0.001 for ASD
IR	Not reported	Not reported
Length of stay	1–2 days: 38/100 (38%) 3–4 days: 21/100 (21%) 5–7 days: 30/100 (30%) >7days: 11/100 (11%)	Not reported
PICU admission	10/100 (10%) PICU admission p=0.426 for clinically significant PTSD	Not reported
Definition of functional outcomes	Not reported	Not reported
Incidence of poor functional outcomes	Not reported	Not reported

ASD, acute stress disorder; IR, interventional radiology; PICU, paediatric intensive care unit; PTSD, post-traumatic stress disorder.

The study by Al Zomia *et al* was conducted at a single children’s hospital in Saudi Arabia.^5^ This study included 100 children, with a mean age of 7.3±3.4 years (SD), who had major orthopaedic trauma, with 70/100 (70%) requiring an operation.^5^ The study by Liu *et al* was undertaken at a single centre in China.^6^ This study included 105 children, with a mean age of 11.8±3.8 years (SD), who had been injured in an earthquake, with 83/105 (79%) requiring an operation. Both studies observed major trauma and operative management to be statistically significantly associated with adverse psychological outcomes compared with those managed conservatively. The included studies were heterogeneous, with a high risk of bias and low confidence in the effect estimate (online supplemental Appendix 1).

## Discussion

This systematic review identified two studies, both of which demonstrated that paediatric major trauma patients who had operative management had a greater risk of adverse psychological outcomes than those managed conservatively. However, the evidence base was small, and significant heterogeneity was found between the two studies, making generalisability and comparisons challenging.

Adverse psychological outcomes are frequently observed in paediatric major trauma patients.^2^ Similarly, children and adolescents who undergo invasive management are recognised to be at increased risk of adverse psychological outcomes.^1^ It is not known to what extent major trauma patients requiring invasive management are at risk of adverse psychological outcomes compared with those managed conservatively. This review has several limitations, including heterogeneity, a small number of studies and a high risk of bias. The primary exposures differ, and the absence of reported Injury Severity Scores or a clear definition of major trauma makes it challenging to determine whether the psychological outcomes are due to the intervention itself or the incident. These studies also draw on specific populations, which may not be generalisable, and confounders such as paediatric intensive care unit stay, death of a family member or the impact of a natural disaster (may have major lasting external psychological outcomes not directly related to the injuries from the incident) are either not recorded or not accounted for. Despite these limitations, the study has the advantage of utilising a thorough, broad and peer-reviewed search strategy.^3^

The results of this review highlight a significant gap in the literature related to the hypothesis that paediatric major trauma patients who also require operative management are at greater risk of adverse psychological outcomes. Future studies would benefit from exploring this question while accounting for key confounding factors. Identifying paediatric major trauma patients at increased risk of adverse psychological outcomes can enhance screening for psychological trauma, help pinpoint those most at risk of adverse outcomes and offer early, targeted, holistic support for this group of patients.

## Supplementary material

10.1136/bmjpo-2025-004450 online supplemental file 1
