# Supplementary material for: Psychological outcomes in paediatric major trauma patients who require invasive management: a systematic review
Source: BMJ Paediatr Open. 2026 Mar 19;10(1):e004450. doi: 10.1136/bmjpo-2025-004450 (PMC13007106; doi:10.1136/bmjpo-2025-004450)
Supplement: Supplementary file 2 [file bmjpo-2025-004450.reviewer_comments.pdf]

PEER REVIEW HISTORY

BMJ Paediatrics Open publishes all reviews undertaken for accepted manuscripts. Reviewers are asked to complete a checklist review form and are provided with free text boxes to elaborate on their assessment. These free text comments are reproduced below.

ARTICLE DETAILS

**TITLE (PROVISIONAL)**	Psychological outcomes in paediatric major trauma patients who require invasive management: a systematic review
**AUTHORS**	Hibberd, Owen<br>Thomas, Caroline<br>Gentle, Sarah<br>Angus, Sandi<br>Karageorgos, Spyridon<br>Thomas, Stephen H

VERSION 1 – REVIEW

**REVIEWER NAME**	Kirk, Angela
**REVIEWER AFFILIATION**	KK Women's and Children's Hospital
**REVIEWER CONFLICT OF INTEREST**	No Conflict of Interest
**AI DISCLOSURE**	No, I have not used AI
**DATE REVIEW RETURNED**	23-Jan-2026

**GENERAL COMMENTS**	Minor comments:<br>1) Mean age (?SD) to define<br>2) To expand the acronyms in the table

**REVIEWER NAME**	Goh, Mark
**REVIEWER AFFILIATION**	
**REVIEWER CONFLICT OF INTEREST**	No Conflict of Interest
**AI DISCLOSURE**	No, I have not used AI
**DATE REVIEW RETURNED**	18-Feb-2025

**GENERAL COMMENTS**	This systematic review address important issues relating to paediatric major trauma and the search strategy appears thorough. However, the evidence base is small (n=2) and the framing of the conclusions exceed what these studies can reasonably support. There are conceptual issues regarding the exposure definition, in particular the natural disaster setting, which may have additional confounders. The authors may have overstated their findings based on the given evidence. The manuscript could instead focus on highlighting a gap in evidence rather than implying an association.<br><br>1. Primary exposure<br>- The primary exposures are quite different, there is no reported injury severity score (ISS) and hence it makes it very challenging if the psychological outcomes are due to the intervention itself or due to the incident incident (where the injury severity can be far more heterogenous in an earthquake)

	- No clear definition of major trauma<br>- Perhaps the authors can try to further group the severity of the injuries and hence a better association with intervention/conservative treatment and the psychological outcomes<br><br>2. Heterogeneity of studies<br>- both studies are very different, making generalizability and comparisons challenging e.g. region, incident, assessment outcomes<br>- mass trauma in earthquakes may have major lasting external psychological outcomes not directly related to the incident e.g. loss of housing, family members, which may outweigh the perceived psychological impact from intervention<br><br>3. Discussion<br>- the authors are honest about the limitations of the study and include a strength as a thorough, broad, and peer-reviewed search strategy<br>- the authors mention "Both studies were at high risk of bias and had low confidence in the effect estimate" but the language in "The results of this review support (and should stimulate further evaluation of) the hypothesis that paediatric major trauma patients who also require operative management are at greater risk of adverse psychological outcomes." is not consistent in tone.<br>- rather than concluding that the review supports the hypothesis (of which the association is not qualitatively strong) they can focus on the heterogeneity of evidence presented, the gaps in the literature, and the difficulties in addressing the hypothesis

VERSION 1 – AUTHOR RESPONSE

Reviewer 1

Ms. Angela Kirk, KK Women's and Children's Hospital

Minor comments:

1) Mean age (?SD) to define

2) To expand the acronyms in the table

Thank you, we really appreciate you noticing this and have updated the table to include an explanation of all acronyms (including Standard Deviation).

Reviewer 2

Mark Goh

This systematic review address important issues relating to paediatric major trauma and the search strategy appears thorough. However, the evidence base is small (n=2) and the framing of the

conclusions exceed what these studies can reasonably support. There are conceptual issues regarding the exposure definition, in particular the natural disaster setting, which may have additional confounders. The authors may have overstated their findings based on the given evidence. The manuscript could instead focus on highlighting a gap in evidence rather than implying an association.

Thank you. We are grateful for this comment and agree. The discussion section of the manuscript has been reframed to highlight this as a gap in the evidence. This is demonstrated in full below with changes highlighted in red.

This systematic review identified two studies, both of which demonstrated that paediatric major trauma patients who had operative management had a greater risk of adverse psychological outcomes than those managed conservatively. However, the evidence base was small, and there was significant heterogeneity between the two studies, making generalisability and comparisons challenging.

Adverse psychological outcomes are frequently observed in paediatric patients with major trauma.[2] Similarly, children and adolescents who undergo invasive management are recognised to be at increased risk of adverse psychological outcomes.[1] It is not known to what extent major trauma patients requiring invasive management are at risk of adverse psychological outcomes compared with those managed conservatively. There are several limitations to this review, including heterogeneity, a small number of studies and a high risk of bias. The primary exposures differ, and the absence of reported Injury Severity Scores or a clear definition of major trauma makes it challenging to determine whether the psychological outcomes are due to the intervention itself or the incident. These studies also draw on specific populations, which may not be generalisable, and confounders such as PICU stay, death of a family member, or the impact of a natural disaster (may have major lasting external psychological outcomes not directly related to the injuries from the incident) are either not recorded or not accounted for. However, despite these limitations, the study has the advantage of utilising a thorough, broad, and peer-reviewed search strategy.[3]

The results of this review highlight a significant gap in the literature related to the hypothesis that paediatric major trauma patients who also require operative management are at greater risk of adverse psychological outcomes. Future studies would benefit from exploring this question while accounting for key confounding factors. By identifying paediatric major trauma patients at increased risk of adverse psychological outcomes, we can enhance screening for psychological trauma, help pinpoint those most at risk of adverse outcomes, and offer early, targeted, holistic support for this group of patients.

---

1. Primary exposure

- The primary exposures are quite different, there is no reported injury severity score (ISS) and hence it makes it very challenging if the psychological outcomes are due to the intervention itself or due to the incident incident (where the injury severity can be far more heterogenous in an earthquake)
- No clear definition of major trauma
- Perhaps the authors can try to further group the severity of the injuries and hence a better association with intervention/conservative treatment and the psychological outcomes

Thank you. We agree with all these points and have updated the manuscript accordingly. The two studies included are limited and do not allow further subgrouping by injury severity.

2. Heterogeneity of studies

- both studies are very different, making generalizability and comparisons challenging e.g. region, incident, assessment outcomes
- mass trauma in earthquakes may have major lasting external psychological outcomes not directly related to the incident e.g. loss of housing, family members, which may outweigh the perceived psychological impact from intervention

Thank you. We agree with all of these points and have updated the manuscript accordingly.

3. Discussion

- the authors are honest about the limitations of the study and include a strength as a thorough, broad, and peer-reviewed search strategy
- the authors mention "Both studies were at high risk of bias and had low confidence in the effect estimate" but the language in "The results of this review support (and should stimulate further evaluation of) the hypothesis that paediatric major trauma patients who also require operative management are at greater risk of adverse psychological outcomes." is not consistent in tone.
- rather than concluding that the review supports the hypothesis (of which the association is not qualitatively strong) they can focus on the heterogeneity of evidence presented, the gaps in the literature, and the difficulties in addressing the hypothesis

Thank you. We agree with all of these points and have updated the manuscript accordingly.

VERSION 2 – REVIEW

**REVIEWER NAME**	Goh, Mark
**REVIEWER AFFILIATION**	
**REVIEWER CONFLICT OF INTEREST**	No Conflict of Interest
**AI DISCLOSURE**	No, I have not used AI
**DATE REVIEW RETURNED**	04-Mar-2026

**GENERAL COMMENTS**	Accepted changes to manuscript
